# Huygens synchronization of two clocks

**DOI:** 10.1038/srep11548

**Published:** 2015-07-23

**Authors:** Henrique M. Oliveira, Luís V. Melo

**Affiliations:** 1 Center of Mathematical Analysis Geometry and Dynamical Systems, Department of Mathematics, Técnico, Universidade de Lisboa, Av. Rovisco Pais, 1049-001, Lisboa, Portugal; 2 INESC-MN and IN, Av. Alves Redol 9, 1000-029 Lisboa, Portugal and Department of Physics, Técnico, Universidade de Lisboa, Av. Rovisco Pais, 1049-001, Lisboa, Portugal

## Abstract

The synchronization of two pendulum clocks hanging from a wall was first observed by Huygens during the XVII century. This type of synchronization is observed in other areas, and is fundamentally different from the problem of two clocks hanging from a moveable base. We present a model explaining the phase opposition synchronization of two pendulum clocks in those conditions. The predicted behaviour is observed experimentally, validating the model.

The synchronization between two periodic systems connected through some form of coupling is a recurrent, and still pertinent, problem in Nature, and in particular in Physics. During the 17th century Huygens, the inventor of the pendulum clock, observed phase or phase opposition coupling between two heavy pendulum clocks hanging either from a house beam and later from a board sitting on two chairs^1^. These two systems are inherently different in terms of the coupling process, and in consequence of the underlying model. The later case has been thoroughly studied^2^,^3^,^4^,^5^,^6^,^7^,^8^,^9^ by considering momentum conservation in the clocks-beam system. The first case has been approached in theoretical works^10^,^11^,^12^,^13^. We present a mathematical model where the coupling is assumed to be attained through the exchange of impacts between the oscillators (clocks). This model presents the additional advantage of being independent of the physical nature of the oscillators, and thus can be used in other oscillator systems where synchronization and phase locking has been observed^14^.The model presented starts from the Andronov^15^ model of the phase-space limit cycle of isolated pendulum clocks and assumes the exchange of single *impacts* (sound solitons, for this system) between the two clocks at a specific point of the limit cycle. Two coupling states are obtained, near phase and near phase opposition, the latter being stable. Our experimental data, obtained using a pair of similar pendulum clocks hanging from an aluminum rail fixed to a masonry wall, match the theoretical predictions and simulations.

## Andronov model

The model for the isolated pendulum clock has been studied using models with viscous friction by physicists^2^,^3^,^5^,^6^,^7^,^8^,^9^. However, Russian mathematicians lead by Andronov published a work^15^ where the stability of the model with dry friction is established (*Andronov clock)*. The authors prove the existence and stability of the limit cycle.

**Figure 1:**
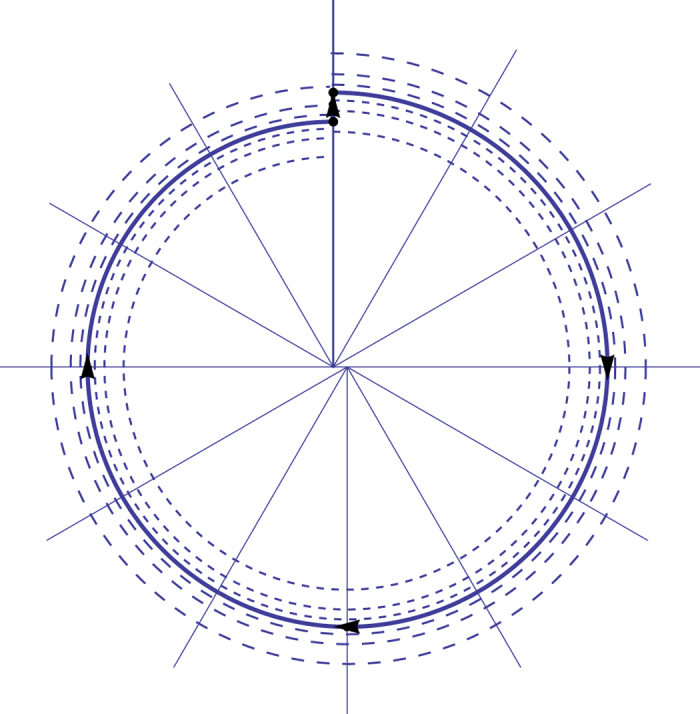
Limit cycle of an isolated clock represented as a solid curve in the phase space. Horizontal axis represents the angular position and in the vertical axis the velocity. We use normalized coordinates to get arcs of circles.

We adopt as basis for our work the first of the aforementioned models, assuming that dry friction predominates. Using the angular coordinate *q*, the differential equation governing the pendulum clock is


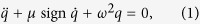


where *μ* > 0 is the dry friction coefficient, *ω* is the natural angular frequency of the pendulum and sign(*x*) a function giving −1 for *x* < 0 and 1 for *x* > 0 and sign 0 ∈ [−1,1]. In^15^ was considered that, in each cycle, a fixed amount of normalized kinetic energy 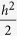 is given by the escape mechanism to the pendulum to compensate the loss of kinetic energy due to dry friction in each complete cycle. We call to the transfer of kinetic energy a *kick*. We set the origin such that the kick is given when 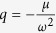, which is very close to 0. The phase portrait is shown in Fig. 1.

There are anchors with geometries allowings two symmetric kicks per cycle. The theoretical treatment is similar but we adopted the first model, with one kick per cycle, due to the geometry of the anchor of the clocks used in the experimental setting.

Considering initial conditions 
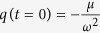
 and 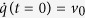, we draw a Poincaré section (16 vol. II, page 268) as the half line 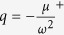 and 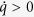 15. The symbol + refers to the fact that we are considering that the section is taken immediately after the kick. There is a loss of velocity 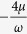 due to friction during a complete cycle. Considering 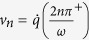 the velocity at the Poincaré section in each cycle one obtains^15^ the non-linear discrete dynamical system


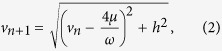


which has the asymptotically stable fixed point


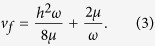


The fixed point (2) attracts initial conditions *v*_0_ in the interval 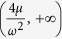.

## Model for two pendulum clocks

We consider two pendulum clocks suspended at the same wall. When one clock receives the kick, the impact propagates in the wall slightly perturbing the second clock. The perturbation is assumed instantaneous since the time of travel of sound in the wall between the clocks is assumed very small compared to the period. The interaction was studied geometrically and qualitatively by Abraham^10^,^11^. However, that approach does not give estimates on the speed of convergence.

In Vassalo-Pereira^13^, the theoretical problem of the phase locking is tackled. The author makes the assumptions:

1. Dry friction.
2. The pendulums have the same exact natural frequency *ω*.
3. The perturbation in the momentum is always in the same vertical direction in the phase space, see also^10^,^11^.
4. The perturbation imposes a discontinuity in the momentum but not a discontinuity in the dynamic variable.
5. The interaction between clocks takes the form of a Fourier series^12^.

Vassalo-Pereira deduced that the two clocks synchronize with zero phase difference. This is the exact opposite of Huygens first remarks^1^ and our experimental observations, where phase opposition was observed. Therefore, we propose here a modified model accounting for a difference in frequency between the two clocks.

Consider two oscillators indexed by *i* = 1,2. Each oscillator satisfies the differential equation


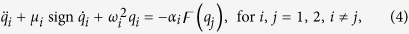


when 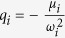, the kinetic energy of each oscillator is increased by the fixed amount *h*_*i*_ as in the *Andronov model*. The coupling term is the normalized force 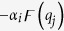, where 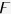 is the interaction function and *α*_*i*_ a constant with acceleration dimensions.

We consider that the effect of the interaction function 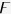 is to produce an increment −*α* in the velocity of each clock leaving the position invariant when the other is struck by the energy kick, as we will see in equations (9). We could consider that the interaction function is the Dirac delta distribution 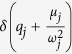, giving exactly the same result.

The sectional solutions of the differential equation (4) are obtainable when the clocks do not suffer kicks. To treat the effect of the kicks we construct a discrete dynamical system for the phase difference. The idea is similar to the construction of a Poincaré section. If there exists an attracting fixed point for that dynamical system, the phase locking occurs.

Our assumptions are

1. Dry friction.
2. The pendulums have natural angular frequencies *ω*_1_ and *ω*_2_ near each other with *ω*_1_ = *ω* + *ε* and *ω*_2_ = *ω* − *ε*, where *ε* ≥ 0 is a small parameter.
3. The perturbation in the momentum is always in the same vertical direction in the phase space^10^,^11^.
4. Since the clocks have the same construction, the energy dissipated at each cycle of the two clocks is the same, *h*_1_ = *h*_2_ = *h*. The friction coefficient is the same for both clocks, *μ*_1_ = *μ*_2_ = *μ*.
5. The perturbative interaction is instantaneous. This is a reasonable assumption, since in general the perturbation propagation time between the two clocks is several orders of magnitude lower than the periods.
6. The interaction is symmetric, the coupling has the same constant *α* when the clock 1 acts on clock 2 and conversely. In our model we assume that *α* is very small.

All values throughout the paper are in SI units when not explicit.

**Figure 2:**
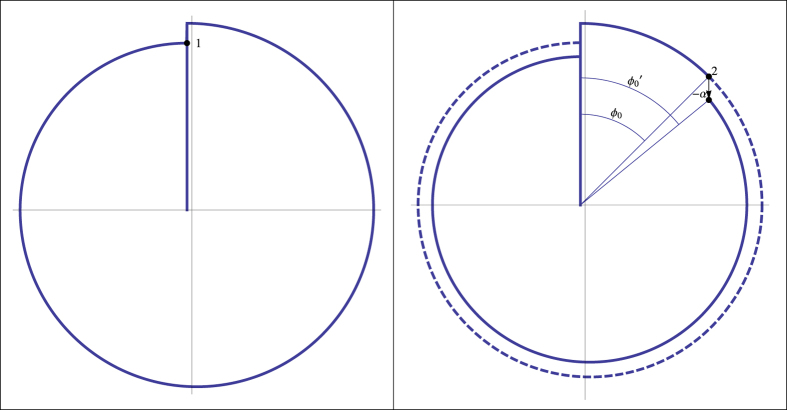
Interaction of clock 1 on clock 2 at *t* = 0^+^. We see the original limit cycle, before interaction, and the new one in solid and the original limit cycle in dashed. Note that the value of *α* and of *h* are greatly exaggerated to provide a clear view. The effect of the perturbation is secular and cumulative.

**Figure 3:**
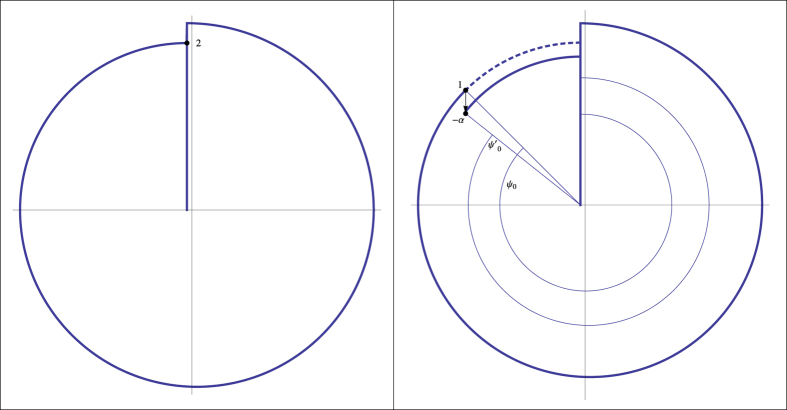
Second interaction. Interaction of clock 2 on clock 1 when clock 2 reaches its impact position. All the features are similar to the Fig. 2.

To prove phase locking we solve sectionally the differential equations (4) with the two small interactions. Then, we construct a discrete dynamical system taking into account the two interactions per cycle seen in Fig. 2 and 3. After that, we compute the phase difference when clock 1 returns to the initial position. The secular repetition of perturbations leads the system to near phase opposition as we can see by the geometrical analysis of Fig. 2 and 3.

The notation is simplified if we consider the function *γ*(*φ*) such that


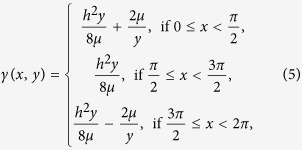


and the function *χ*(*φ*) such that


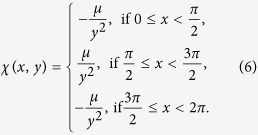


We make the assumption that the natural frequencies are near. A difference of 28 s per day in the movement of the clocks with natural periods in the order of 1.42 s, which is easy to obtain even with very poor clocks, means that *ε* is on the order of 10^−3^rads^−1^.

This means that, in each cycle of each clock, the other one will give one perturbative kick to the other. Suppose that the clocks are bring to contact at *t*_0_ = 0. Consider that the fastest clock (number 1) is at position


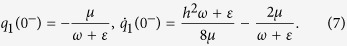


Using *γ* and *χ* we have


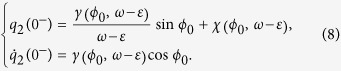


The perturbation of clock 1 on clock 2 adds the value of −*α* to the velocity 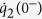, keeping the position *q*_2_(0^−^).Thus, the new initial conditions at *t* = 0^+^ for the movement of the second clock are


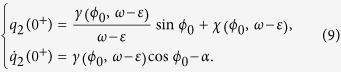


The new phase of clock 2, which is the phase difference of the two clocks *φ*′_0_, at 0^+^ is now


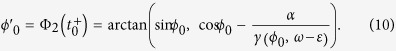


To simplify the notation we consider the function


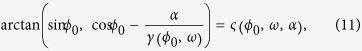


therefore


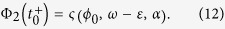


With power expansion in *α*


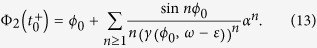


The correction of the phase difference at 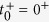 is


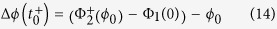



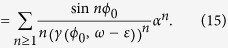


With first order term in *α*


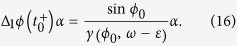


Now, both clocks start their natural movement.

We suppose that the clock 2 arrives at the vertical position without being overtaken by clock 1, if that is the case we begin our study after that situation occurs. Clock 2 takes the time 
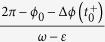
 to arrive at this position. The phase of clock 2 is 
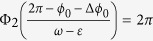
. The phase of clock 1 is now


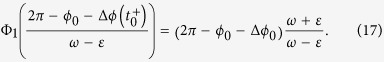


The next interaction is given by the kick from clock 2 to clock 1. Denoting the phase of clock 1 at this stage by


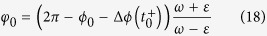


and 
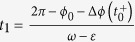
, we have the phase difference immediately before the second kick


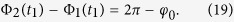


The next interaction is given by the kick from clock 2 to clock 1. Using a process similar to the previous kick we have after the second kick


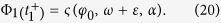


Expanding this function in power series we have


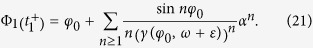


The new phase difference is


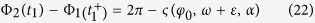



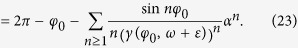


The correction of the phase difference at 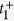 is now


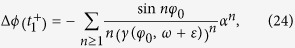


with first order term in *α*


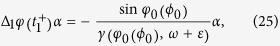


and this expression can be further simplified remembering that


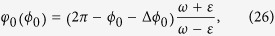


giving in first order in *α*


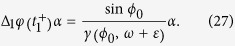


To complete the study of the phase difference the clock 1 must return to the vertical position, which happens for the time 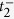. The time that this clock takes to return to the vertical position is 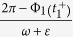, the phase of clock 1 is now 2*π*, the phase of clock 2 gives us the new phase difference *φ*_1_ after one cycle of clock 1, that is


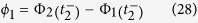



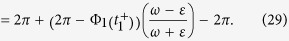



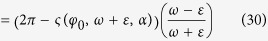



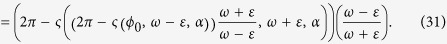


If we call the affine function


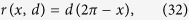


and the coefficients


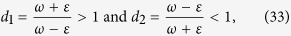


the large expression (31) is the composition of four maps


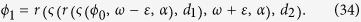


The discrete dynamical system for *n* ≥ 0 is given by the map Ω such that


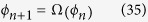



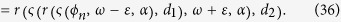


Obviously, Ω is a map from the interval [0,2*π*] to itself. Despite the apparent complexity of Ω, this map is relatively manageable. Under certain conditions we can prove that Ω has a stable fixed point. In this work we deal only with the first degree approximation, relative to the small parameters *α* and *ε*, the value of the fixed point *φ*_*f*_ which is near to *π*. The phase difference is asymptotic to the solution *φ*_*f*_. Knowing this value it is possible to prove the existence and stability of the limit cycle of each clock in interaction and the final asymptotic frequency *ω*_*f*_. Under this model we can say that Huygens sympathy occurs.

In first order of *α* and *ε* we have the iterative scheme


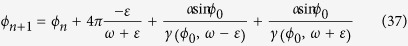



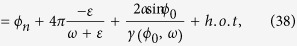


We define the map


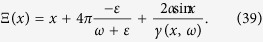


We get in first order of *α* and *ε* the dynamical system for the phase difference


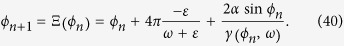


There are two fixed points 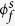 and 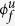 of Ξ in the interval [0,2*π*]


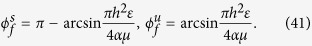


The derivative of Ξ at the fixed point 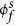 must be |Ξ′(*x*_*f*_)| < 1 to have stability and the condition about the argument of the function arcsin gives


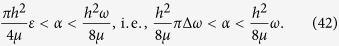


Therefore, the limit of the phase difference is, in first order, 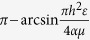 which is very near to *π* when the natural frequencies of both clocks are very near, i.e., small *ε*. When the system reaches this limit the corrections of phase are null for both clocks.

## Simulation

To study the Huygens synchronization we used numerical simulations. We applied the map Ξ(*x*) without performing the Taylor expansion. We used the environment of Wolfram Mathematica 9.0 to produce the computations. The values of *μ*, *h*, *ω*, *t*_0_ were taken realistically from the experimental setup and kept fixed throughout the simulations. The coupling constant *α* and the half-difference between the clocks frequencies *ε* are adjusted in simulations.

Additionally, we introduced noise in the model with normal distribution acting directly on the phase. The effect of noise is to mimic the small perturbations that occur in the lab, e.g., vibrations in the wall and the stochastic changes of the level of the interaction, cycle after cycle. The strength of this stochastic effect is given by the parameter *ρ*. When the noise function is not used, i.e. *ρ* = 0, if the parameters are in the convergence region given by conditions (42) we have a fixed convergence point of Ω.

## Experimental

**Figure 4:**
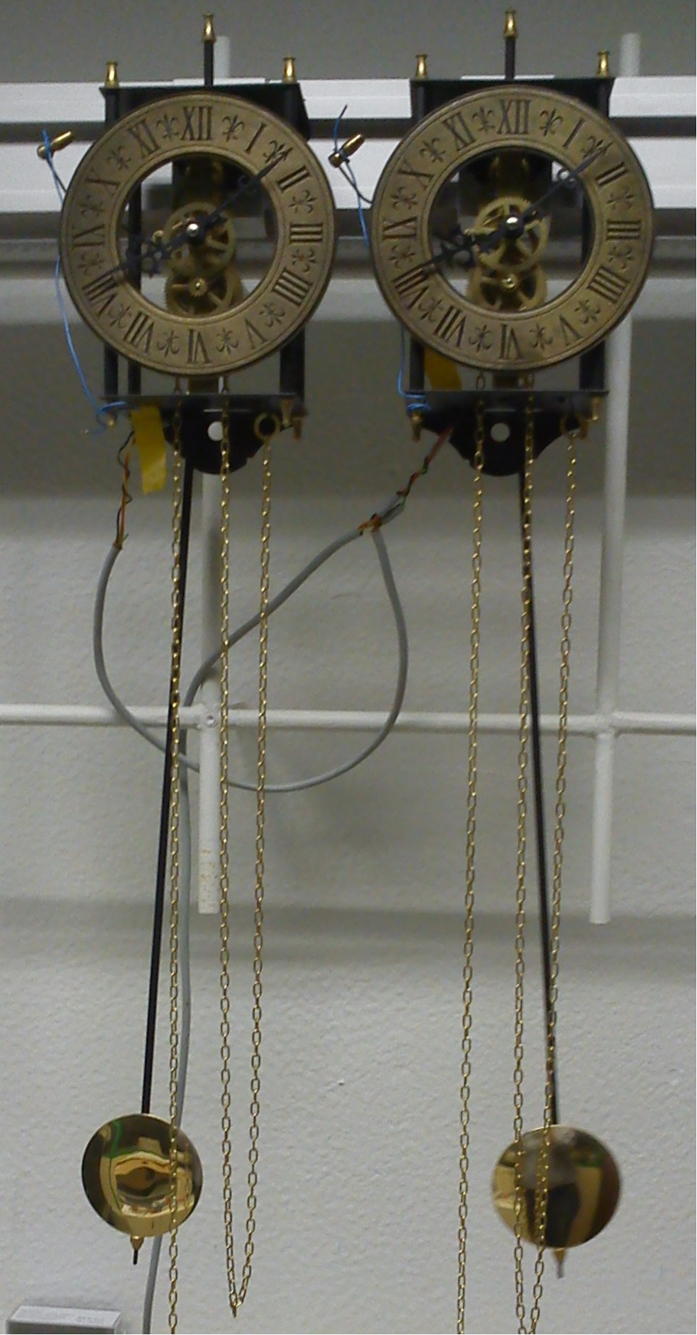
Photo of the experimental setup. The clocks can be seen hanging from the Al rail. The weights are outside of the picture. The cables in the background connect the optical sensors.

Experiments were setup using a standard optical rail (Eurofysica) rigidly attached to a wall to which two similar pendulum clocks were fixed through modified rail carriers. This setup can be seen in Fig. 4. The clocks were 230 mm apart. This was the lowest distance still ensuring that the pendulums trajectories were completely separated. The mass-driven anchor-pendulum clocks used were Acctim 26268 Hatahaway. One mass travel supplies energy for around 5 days, and the clocks take about one day to relax to the final frequency after winding. The period, of the order of 1.42 s, is configured by rotating the screw at the bottom of the pendulum, thus changing the actual pendulum length. The chime mechanism was inhibited in order to reduce mechanical noise. Time was measured using one U-shaped LED emitter-receptor TCST 1103, connected to a Velleman K8055 USB data acquisition board operated with custom-developed Visual Basic 6 software running in a standard personal computer (PC). The time measured corresponds to the left maximum angle, and is the midpoint of the interval of the sensor coverage by the pendulum beam. The uncertainty in time acquisition typically expected in the ms range for the PC was overcame by performing running averaging of the period data, up to 1000. The data files were then processed offline using Mathematica. In order to obtain the appropriate parameters for the simulation, the pendulums were filmed and the movement quantified using free software *Tracker4.84* (last date of access: 27/03/2015) from OSP https://www.cabrillo.edu/~tracker.

We assume that the coupling is obtained through the exchange of sound pulses between the clock propagated through the rail. This is consistent with the absence of coupling for other materials tried (MDF and fiberglass) The exact mechanism of how the pulse energy propagates through the clock hardware down to the pendulum is hard to assess in detail and depends on the individual clock. The sound propagation speed in Al is 6420 ms^−1^, leading to a propagation time of the order of 3.0 × 10^−5^ s, which is negligible in face of the 1.42 s period of the oscillators, and so the instantaneous propagation of sound assumed in the model is a reasonable approximation.

## Results and discussion

Observing the movement of the pendulum alone (as a damped oscillator; initially at the limit cycle), we noticed a decrease of the maximum velocity of the pendulum according to a linear fit *v*_max_ = 0.2228 − 0.0023*n*, where *n* is the number of cycles, with correlation coefficient 0.994. The decrease of velocity per cycle predicted by the Andronov model is 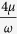. With the value of the period *T* = 1.40000s and 
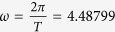
 rad/s, we can estimate the value of 
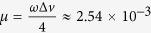
.

The value of *h* can also be easily established by studying the movement at the limit cycle. We then have the maximum velocity 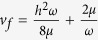. We found consistently that the maximum velocity at the limit cycle is *v*_*f*_ = 0.223 m/s. Therefore, *h* ≈ 0.032.

We used different possible values of natural frequency difference. For the average natural angular frequency we take the same value of *T* = 1.40000 s, hence *ω* = 4.48780 rad/s. The fastest clock has a natural frequency of *ω* + *ε* and the slowest *ω* − *ε*. The angular frequency difference is in first order 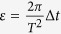, where Δ*t* is half of the period difference. Notice that when Δ*t* = 2 × 10^−4^s, with a delay between the clocks of 24.6 s per day for the non coupled pair of clocks, the value of *ε* is *ε* = 6.4 × 10^−4^ rad/s. We used values of *ε* in the range 10^−4^ to 10^−3^ rad/s as a realistic estimate for the performance of our setup.

The fixed parameters used for the simulations are then *μ* = 2.54 × 10^−3^, *h* = 0.032, *ω* = 4.48799 rad/s, *t*_0_ = 0.8*π* s, and the phenomenological noise coefficient of *ρ* = 0.093, which fits the ripple observed at the experimental data. When we choose *ε* = 3 × 10^−3^ rad/s, corresponding to a huge natural delay of 116 s per day with the clocks in the isolated state, a value of *α* = 7 × 10^−4^ yielded results matching the experimental data. The conditions (42), using the linear approximation, give a estimate threshold of *ε* = 2.2 × 10^−3^ rad/s for the coupling with the same values of the fixed parameters. This is not contradictory with our results, since we used in the simulations the function Ξ with no approximations and we found numerically that the actual threshold for that function is at *ε* = 3.5 × 10^−3^ rad/s.

**Figure 5:**
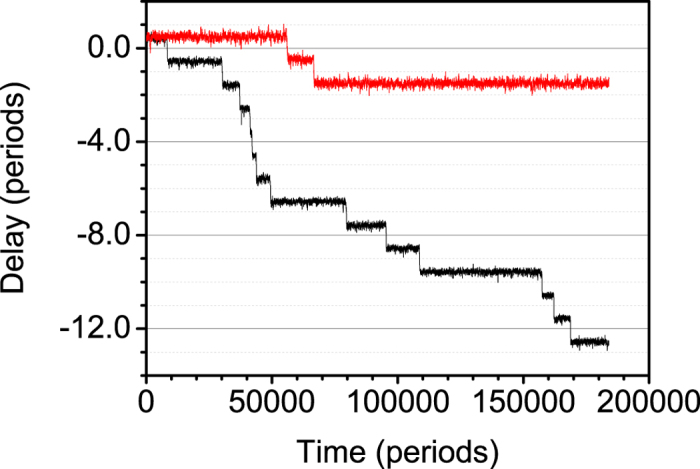
Simulations of delay between the two clocks in period units for two frequency differences. Upper curve (red): *ε* = 1.5 × 10^−4^ rad/s. Lower curve (black): *ε* = 3.0 × 10^−3^ rad/s. *ω* = 4.48799 rad/s.

We expect more frequent escapes from stable states than if we choose *ε* = 1.5 × 10^−4^ rad/s, corresponding to a natural delay of 2.9 s per day. These values correspond to the values that could realistically be obtained in our experimental setup. The plots can be seen in Fig. 5.

Notice the small differences assumed for the frequencies, of the same order of the values measured for independent clocks. The time difference stabilizes in horizontal plateaus, corresponding to phase opposition coupling. The stochastic term introduced in the simulation unsets the system at some point, and then the phase difference increases quickly as the fastest clock runs away until the next synchronization plateau is reached, one or sometimes two periods away. For the simulation with the smaller difference between frequencies, the number of transitions between plateaus is smaller, as expected since the stability is much easier to reach and maintain.

**Figure 6:**
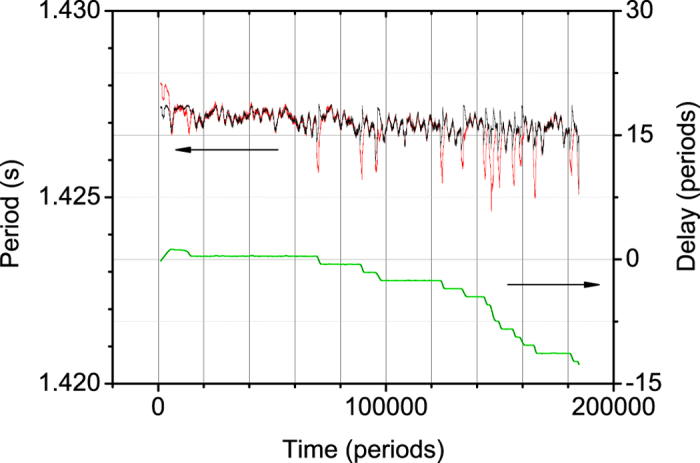
Phase difference between the clocks in period units over more than three days (lower curve in green, right axis) and periods of the two clocks (upper curves, left axis; red for clock 1, black for clock 2). The initial behaviour corresponds to mechanical stabilization of the clocks during the first few hours of the experiment.

This is strikingly similar to the behaviour observed in Fig. 6 for the actual clocks (right axis). The number of synchronization plateaus is of the same order and can be fine-tuned using the stochastic parameter in the simulation. It was observed that the system could be unsettled by a number of external noise sources, e.g. from doors closing nearby in the building, people entering or leaving the room, or even the elevator stopping, than proceeding to the next synchronization plateau.

The periods of both clocks from the same experiment can be seen on the same figure (left axis). The periods vary together within an interval of about 1 ms around 1.427 s when the clocks are coupled with correlation coefficient above 0.97 in the coupled state. Notice the almost perfect coincidence of the two curves except when the system leaves coupled states. Notice also the unstability of the coupled period, varying over an interval of almost 1 ms. When coupling is lost the period of one clock decreases sharply (up to 2 ms or more) and the period of the other clock increases by a (smaller) amount. These perturbations in the periods are coincident with the loss of phase opposition coupling. Although one may expect changes in the frequency even when the clocks are not coupled, due to the interaction between them, the difference in period can be estimated at around 2 ms, corresponding to a difference in frequency of the order of 6 × 10^−3^ rad/s. This asymmetry of the coupled period relative to the original periods is predicted by the model. Both periods return to the previous baseline value when the coupling is restored.

**Figure 7:**
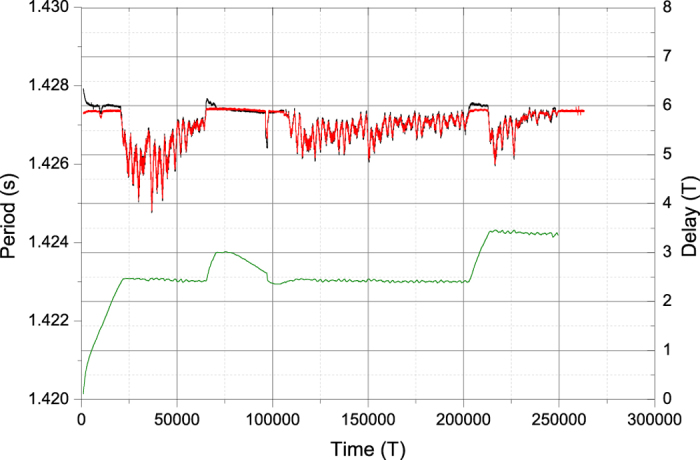
Phase difference between the clocks in period units over more than three days (lower curve in green, right axis) and periods of the two clocks (upper curves, left axis; red for clock 1, black for clock 2). The initial behaviour corresponds to mechanical stabilization of the clocks during the firs few hours of the experiment. After running for around 250000 periods, clock 2 is stopped, and clock 1 keeps running undisturbed.

Fig. 7 shows data for another experiment. In this case the free clock frequencies were closer. Both periods are remarkably coincident, but vary in an interval in excess of 10 ms, when the clocks are coupled. Since the frequencies are closer, the synchronization should be easier to maintain, hence the low number of plateaus, but also should be slower to attain, hence the longer transitions between plateaus. If the perturbation is large enough, especially if the frequencies are very close, it is possible to attain plateaus both above and below. Between *t* = 100000 *T* and *t* = 112000 *T* approximately the synchronization is lost, and the periods become separated by more than 100 *μs* (corresponding to frequency difference around 3 × 10^−4^ rad/s), but become stable (within 1 *s*). At instant *t* = 148000 *T* approximately clock #2 is stopped. From that moment on the period of the remaining working clock becomes stable within about 10 *s*, an interval one order of magnitude below. This confirms that the clocks strongly disturb one another, but also that both periods are kept at the same value in order to keep the synchronization at the expense of some frequency unstability.

## Conclusions

We have developed a model explaining the Huygens problem of synchronization between two clocks hanging from a wall. In this model each clock transmits once per cycle a sound pulse that is translated in a pendulum speed change. An equilibrium situation is obtained for almost half-cycle phase difference. These predictions match remarkably the experimental data obtained for two similar clocks hanging from a wall.

## Additional Information

**How to cite this article**: Oliveira, H. M. and Melo, L. V. Huygens synchronization of two clocks. *Sci. Rep.* **5**, 11548; doi: 10.1038/srep11548 (2015).
